# Delivering gene therapy for mucopolysaccharide diseases

**DOI:** 10.3389/fmolb.2022.965089

**Published:** 2022-09-12

**Authors:** Shaun R. Wood, Brian W. Bigger

**Affiliations:** Stem Cell and Neurotherapies Group, School of Biological Sciences, Faculty of Biology, Medicine and Health, The University of Manchester, Manchester, United Kingdom

**Keywords:** mucopolysaccharidosis, gene therapy, gene editing, vectors, clinical trials

## Abstract

Mucopolysaccharide diseases are a group of paediatric inherited lysosomal storage diseases that are caused by enzyme deficiencies, leading to a build-up of glycosaminoglycans (GAGs) throughout the body. Patients have severely shortened lifespans with a wide range of symptoms including inflammation, bone and joint, cardiac, respiratory and neurological disease. Current treatment approaches for MPS disorders revolve around two main strategies. Enzyme replacement therapy (ERT) is efficacious in treating somatic symptoms but its effect is limited for neurological functions. Haematopoietic stem cell transplant (HSCT) has the potential to cross the BBB through monocyte trafficking, however delivered enzyme doses limit its use almost exclusively to MPSI Hurler. Gene therapy is an emerging therapeutic strategy for the treatment of MPS disease. In this review, we will discuss the various vectors that are being utilised for gene therapy in MPS as well as some of the most recent gene-editing approaches undergoing pre-clinical and clinical development.

## Introduction

The mucopolysaccharidoses (MPS) consist of 11 inherited paediatric lysosomal storage diseases (LSDs) caused by deficiencies in enzymes involved in the breakdown of glycosaminoglycans (GAGs) in lysosomes of the cell (Clarke, 2008). The majority of these conditions (MPSI, MPSIIIA, MPSIIIB, MPSIIIC, MPSIIID, MPSIVA and B, MPSVI, MPSVII and the very rare MPSIX) are inherited in an autosomal recessive manner, while MPSII is X-linked recessive (Celik et al., 2021). Common symptoms seen in MPS conditions include hepatosplenomegaly, skeletal malformations, ocular abnormalities, cardiorespiratory complications, upper airway respiratory infections, hearing loss and, in many cases, central nervous system (CNS) degeneration. The symptoms differ between conditions and depend, to a degree, on the GAG that accumulates. Diseases with predominant heparan sulfate (HS) storage tend to show CNS involvement (MPSI, MPSII, MPSIIIA-D and MPSVII) whereas dermatan sulfate (DS) and keratan sulfate (KS) accumulation leads to more skeletal disease (MPSIVA-B and MPSVI) (Wraith, 2002). These are summarised in Table 1. Enzyme replacement therapy (ERT) has been approved for several MPS conditions such as MPSI (Aldurazyme®, Biomarin, USA), MPSII (Elaprase®, Shire, USA), MPSIVA (Vimizim®, Biomarin, USA), MPSVI (Naglazyme®, Biomarin, USA) and MPSVII (MEPSEVII™, Ultragenyx, USA). ERT has shown some efficacy at treating the non-neurological symptoms of MPS however there are some, such as the skeletal dysplasia seen in many MPS patients that remain poorly corrected. Furthermore, ERT is ineffective in treating the CNS as the blood-brain-barrier (BBB) prevents the movement of large enzymes from the bloodstream into the brain (Wraith, 2006). The only other treatment is haematopoietic stem cell transplant (HSCT) which involves the transplant of matched donor bone marrow or HLA-matched unrelated cord blood units (Valayannopoulos and Wijburg, 2011; Noh and Lee, 2014). Following myeloablative conditioning, donor-derived macrophage cells from the transplant can traffic to the brain, cross the BBB, secrete the enzyme and cross-correct the condition (Bigger and Wynn, 2014). This approach has shown efficacy in MPSI, where the level of enzyme secretion and overall levels appear adequate to relieve GAG accumulation, however it fails to provide relief for CNS manifestations in other MPS conditions, possibly due to insufficient enzyme expressed from the endogenous promoter in allogenic cells (Bigger and Wynn, 2014).

**TABLE 1:** Mucopolysaccharide Diseases, Enzymes, Storage Material and Symptoms. * MPSIX (hyaluronan deficiency)—somatic bone and joint disease, only one case described.

MPS type	Enzyme deficiency	Storage material	Symptoms
MPSI	L-α-Iduronidase (IDUA)	Heparan Sulfate	Cognitive loss (Severe)
			Hepatosplenomegaly
		Dermatan Sulfate	Skeletal Dysplasia
			Cardio-Respiratory Disease
			Hearing Loss
MPSII	Iduronate-2-sulfatase (IDS)	Heparan Sulfate	Cognitive loss (Severe)
			Behavioural dysfunction (Severe)
			Hepatosplenomegaly
		Dermatan Sulfate	Skeletal Dysplasia
			Cardio-Respiratory Disease
					Hearing Loss
MPSIIIA	Sulfamidase (SGSH)	Heparan Sulfate	Cognitive loss
			Behavioural disfunction
			Hearing Loss
MPSIIIB	N-acetylglucosaminidase (NAGLU)	Heparan Sulfate	Cognitive loss
			Behavioural disfunction
			Hearing Loss
MPSIIIC	Acetyl-Coα-glucosaminide acetyltransferase (HGSNAT)	Heparan Sulfate	Cognitive loss
			Behavioural disfunction
			Hearing Loss
MPSIIID	N-acetylglucosamine 6-sulfatase (GNS)	Heparan Sulfate	Cognitive loss
			Behavioural disfunction
			Hearing Loss
MPSIVA	N-acetylgalactosamine 6-sulfatase (GALNS)	Keratan Sulfate	Skeletal Dysplasia
			Cardio-Respiratory Disease
		Chondroitin 6-Sulfate	Hearing Loss
MPSIVB	β-galactosidase (GBL1)	Keratan Sulfate	Skeletal Dysplasia
			Cardio-Respiratory Disease
			Hearing Loss
MPSVI	Arylsulfatase B (ARSB)	Dermatan Sulfate	Hepatosplenomegaly
			Skeletal Dysplasia
			Cardio-Respiratory Disease
			Hearing Loss
MPSVII	Β-glucuronidase (GUSB)	Heparan Sulfate	Cognitive loss
			Hepatosplenomegaly
		Chondroitin 6-Sulfate	Skeletal Dysplasia
		Dermatan Sulfate	Cardio-Respiratory Disease
			Hearing Loss

Both ERT and HSCT rely on delivery of exogenous enzyme at supra-physiological levels. This is typically required as enzyme is taken up by cells through mannose-6-phosphate (M6P) receptor-mediated uptake and the combination of BBB and reduced receptor density on some cell types can limit the impact of these therapies. ERT is inconvenient to patients due to regular (weekly or biweekly) infusions. ERT can also lead to antibody responses against the enzyme in a proportion of naïve patients, reducing efficacy with repeated infusions (Brooks et al., 2003; Saif et al., 2012; Brands et al., 2013; Langereis et al., 2015). In recent years, gene therapy has risen to the forefront of research into MPS treatment. In this review we will summarise the current pre-clinical and clinical research in this exciting field.

### Gene therapy

Gene therapy involves the modification of genetic information within a cell (either *via* gene replacement or gene editing) with the goal of treating disease. The introduction of genes has been traditionally achieved using viral vectors such as adenovirus (AdV), Adeno-associated virus (AAV) and lentivirus (LV) (Lundstrom, 2018). Non-viral gene delivery has been trialed in the mouse models of MPSI and MPSVII with limited success, therefore this review will predominantly focus on viral delivery. In MPS, research has primarily focused on AAV and LV vectors for the replacement of the relevant transgene. Table 2 lists the current gene therapy clinical trials registered for MPS disease.

**TABLE 2:** List of selected gene therapy clinical trials in MPS diseases.

Clinical trial identifier	Title	Status	Condition	Vector	Delivery	Sponsor	Phase
NCT03580083	RGX-111 Gene Therapy in Patients With MPS I	Ongoing	MPSI	AAV2/9	Intrathecal	Regenexbio	I/II
NCT03488394	Gene Therapy With Modified Autologous Hematopoietic Stem Cells for the Treatment of Patients With Mucopolysaccharidosis Type I, Hurler Variant (TigetT10_MPSIH)	Ongoing	MPSI	LV	HSCGT	IRCCS San Raffaele	I/II
NCT02702115	A Phase I/2, Multileft, Open-label, Single-dose, Dose-ranging Study to Assess the Safety and Tolerability of SB-318, a rAAV2/6-based Gene Transfer in Subjects With Mucopolysaccharidosis I (MPS I)	Ongoing	MPSI	AAV2/6	Intravenous	Sangamo Therapeutics	I/II
				Zinc-Finger Nuclease			
NCT02702115	Ascending Dose Study of Genome Editing by the Zinc Finger Nuclease (ZFN) Therapeutic SB-318 in Subjects With MPS I	Ongoing	MPSI	AAV2/6	Intravenous	Sangamo Therapeutics	I/II
				Zinc-Finger Nuclease			
NCT03566043	RGX-121 Gene Therapy in Patients With MPS II (Hunter Syndrome)	Ongoing	MPSII	AAV2/9	Intra-cerebroventricular	RegenexBio	I/II
NCT04571970	RGX-121 Gene Therapy in Children 5 Years of Age and Over With MPS II (Hunter Syndrome)	Ongoing	MPSII	AAV2/9	Intra-cerebroventricular	RegenexBio	I/II
NCT04597385	Long-term Follow-Up for RGX-121	Ongoing	MPSII	AAV2/9	Intra-cerebroventricular	RegenexBio	I/II
NCT00004454	Phase I/II Study of Retroviral-Mediated Transfer of Iduronate-2-Sulfatase Gene Into Lymphocytes of Patients With Mucopolysaccharidosis II (Mild Hunter Syndrome)	Completed	MPSII	Retrovirus	Intravenous injection of Lymphocytes	Eunice Kennedy Shriver National Institute of Child Health and Human Development (NICHD)/University of Minnesota	I/II
NCT03041324	A Phase I/2, Multileft, Open-label, Single-dose, Dose-ranging Study to Assess the Safety and Tolerability of SB-913, a rAAV2/6-based Gene Transfer in Subjects With Mucopolysaccharidosis II (MPS II)	Ongoing	MPSII	AAV2/6	Intravenous	Sangamo Therapeutics	I/II
				Zinc-Finger Nuclease			
NCT03041324	Ascending Dose Study of Genome Editing by the Zinc Finger Nuclease (ZFN) Therapeutic SB-913 in Subjects With MPS II	Terminated	MPSII	AAV2/6	Intravenous	Sangamo Therapeutics	I/II
				Zinc-Finger Nuclease			
NCT04628871	Long Term Follow-up (LTFU) of Subjects Who Received SB-318, SB-913, or SB-FIX (LTFU)	Ongoing	MPSI	AAV2/6	Intravenous	Sangamo Therapeutics	I/II
			MPSII	Zinc-Finger Nuclease			
NCT01474343	Intracerebral Gene Therapy for Sanfilippo Type A Syndrome	Completed	MPSIIIA	AAVrh10	Intraparenchymal	Lysogene	I/II
NCT02053064	Long-term Follow-up of Sanfilippo Type A Patients Treated by Intracerebral SAF-301 Gene Therapy	Completed	MPSIIIA	AAVrh10	Intracranial	Lysogene	I/II
NCT03612869	Study of AAVrh10-h.SGSH Gene Therapy in Patients With Mucopolysaccharidosis Type IIIA (MPS IIIA) (AAVance)	Ongoing	MPSIIIA	AAVrh10	Intracranial	Lysogene	II/III
2015–000359–26	Phase I/II safety, tolerability and initial efficacy study of adeno-associated viral vector serotype 9 containing human sulfamidase gene after intracerebroventricular administration to patients with MPSIIIA.	Ongoing	MPSIIIA	AAV2/9	Intra-cerebroventricular	Laboratorios del Dr. Esteve, S.A.	I/II
NCT02716246	Phase I/II Gene Transfer Clinical Trial of scAAV9.U1a.hSGSH for Mucopolysaccharidosis (MPS) IIIA	Ongoing	MPSIIIA	AAV2/9	Intravenous	Abeona Therapeutics (ABO-102 now with Ultragenyx)	I/II
NCT04088734	A Phase I/II Open Label, Single-dose, Gene Transfer Study of scAAV9.U1a.hSGSH (ABO-102) in Patients With Middle and Advanced Phases of MPS IIIA Disease	Terminated	MPSIIIA	AAV2/9	Intravenous	Abeona Therapeutics	I/II
NCT04360265	A Long-term Follow-up Study of Patients With MPS IIIA Treated With ABO-102	Ongoing	MPSIIIA	AAV2/9	Intravenous	Abeona Therapeutics	I/II
NCT04201405	Gene Therapy With Modified Autologous Hematopoietic Stem Cells for Patients With Mucopolysaccharidosis Type IIIA	Ongoing	MPSIIIA	LV	HSCGT	Orchard Therapeutics/University of Manchester	I/II
NCT03300453	Intracerebral Gene Therapy in Children With Sanfilippo Type B Syndrome	Completed	MPSIIIB	AAV2/5	Intraparenchymal	Institut Pasteur/UniQure Biopharma B.V.	I/II
NCT03315182	Gene Transfer Clinical Trial for Mucopolysaccharidosis (MPS) IIIB (MPSIIIB)	Terminated	MPSIIIB	AAV2/9	Intravenous	Abeona Therapeutics	I/II
NCT04655911	A Long-term Follow-up Study of Patients With MPS IIIB Treated With ABO-101	Ongoing	MPSIIIB	AAV2/9	Intravenous	Abeona Therapeutics	I/II
NCT03173521	Gene Therapy in Patients With Mucopolysaccharidosis Disease	Ongoing	MPSVI	AAV2/8	Intravenous	Fondazione Telethon	I/II

Gene therapy, when delivered correctly should be able to achieve supra-physiological enzyme levels in all target organs affected by MPS by delivery a continuous supply of enzyme that’s secreted by transduced cells.

### AAV-mediated gene therapy

AAV is a non-enveloped virus, measuring ∼25 nm in diameter and belonging to the parvoviridae family of viruses. AAV contains a relatively small, single-stranded, DNA genome of ∼4.7 kb which, following transduction, exists predominantly episomally within the cell (Choi et al., 2006). It is currently one of the most popular vectors for gene therapy and is currently being used in clinical trials for neurological conditions (Spinal Muscular Atrophy) and inherited retinal disorders (Choroideremia, Leber’s Congenital Amaurosis, X-Linked Retinitis Pigmentosa), amongst others (MacLaren et al., 2014; Fischer et al., 2017; Mendell et al., 2017). This popularity is down to a number of factors. There are a wide-range of serotypes available, each with a broad range of tropisms for different tissue types (Zincarelli et al., 2008; Brown et al., 2021). The main limitations of AAV use are due to the relatively small packaging capacity of about 4.7 kb, that only allows small transgenes to be packaged alongside a suitable promoter and other regulatory sequences, and a delayed onset of gene expression due to the requirement for “second-strand synthesis” after transduction. The latter problem can be resolved by using “self-complementary” or “sc” AAV vectors, where a duplicate of the transgene is packaged in an inverted repeat so that second strand synthesis is not required, but this also approximately halves the packaging capacity of the vector (McCarty, 2008).

**FIGURE 1:**
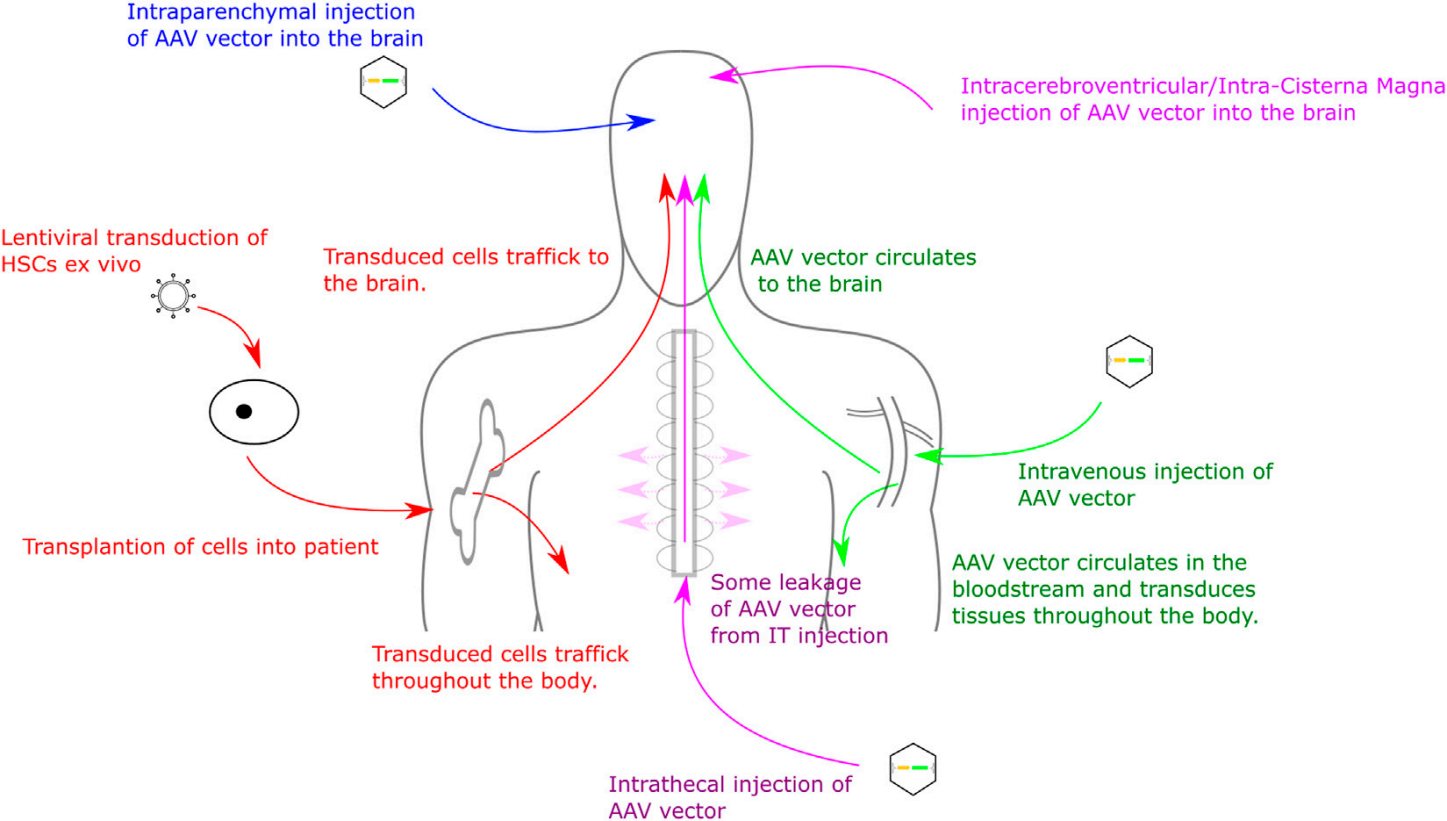
Routes for Gene Therapy Administration to the CNS. Routes into the CNS include direct intracranial delivery, intrathecal delivery, intravenous delivery and haematopoietic stem cell transplant.

Delivery routes for AAV vectors include intracranial injections to the parenchyma of the brain (extensively tested in multiple MPS mouse models) (Cressant et al., 2004; Desmaris et al., 2004; Liu et al., 2007; Tardieu et al., 2014; Winner et al., 2016), intra-CSF injections (intracerebroventricular, intracisternal magna or intrathecal injection) (Karolewski and Wolfe, 2006; Fu et al., 2010; Wolf et al., 2011; Haurigot et al., 2013; Roca et al., 2017) and intravenous, or IV, injection (direct injections into the bloodstream, often with the goal of delivery to the liver to generate high levels of transgene expression in the blood (Hinderer et al., 2014). Intraparenchymal injections can achieve high level expression local to the transduction site and, dependent on AAV serotype, can also achieve expression distal from transduction site (Cearley and Wolfe, 2007; Miyake et al., 2015). Intraparenchymal delivery also typically requires a smaller volume of AAV than IV or intrathecal delivery and demonstrates reduced off-target effects (Haery et al., 2019). Despite this, drawbacks include an invasive surgery that can produce damage local to the injection site and expression distal from injection site is often lower than expression local to the injection site (Carty et al., 2010; Stoica et al., 2013). IV injections are far less invasive than intracranial delivery. Several AAV serotypes (in particular AAV9 and AAVrh10 and a number of new serotypes) have the ability to cross the BBB, often by binding HS on the cell surface, and therefore IV delivery can achieve a more uniform expression in the brain than intracranial delivery (Stoica et al., 2013; Chan et al., 2017; Colella et al., 2018). Drawbacks of this approach are primarily due to the amount of vector needing to be delivered to patients in order to achieve effective transduction of the brain. This volume of vector can be expensive to produce, as well as increasing the potential for an immune response against the vector (Mingozzi and High, 2013). Furthermore, as the vector is delivered into the bloodstream it will inevitably reach more tissues than if injected into the CNS, increasing the likelihood of off-target transduction. Delivery to the CSF includes intrathecal (IT) intracerebroventricular (ICV) or intracisterna magna (ICM) injections. These can provide widespread gene expression throughout the brain and CNS, however expression is often not confined to the CNS and the expression is not as uniform as with IV injections (Gessler et al., 2019). Like IV injections, a relatively large amount of virus is required compared to intracranial delivery. These delivery methods are described in more detail by Haery and colleagues (Haery et al., 2019) and are summarised in Figure 1. Recently, comparisons of ICV, IT and intranasal (IN) delivery of AAV9 in MPSI mice demonstrated increased α-iduronidase (IDUA) activity in the brains of all treated mice compared to untreated controls. Increases were up to 1000-fold of wild-type expression in all areas of the brain *via* ICV injection. IT injections also produced up to 1000-fold of wild-type expression but distribution was less uniform. IN delivery generated up to 100-fold of wild-type expression in the olfactory bulb with expression levels throughout the rest of the brain similar to wild-type (Belur et al., 2021), however IN delivery has not yet been adopted in clinical practice as other groups have been unable to replicate this brain distribution. Of course distribution in non-human primates and in humans could well be somewhat different, so it is important to establish delivery protocols and serotype efficacy in at least two species, one of which should be a large animal model.

### Haematopoietic stem cell gene therapy

Another promising therapeutic approach in MPS is the transplantation of gene-modified haematopoietic stem cells (Holley et al., 2019). Termed “haematopoietic stem cell gene therapy” or “HSCGT,” this procedure involves the re-introduction of the relevant corrected gene into haematopoietic stem cells (HSCs) and their subsequent transplantation into a patient, following chemotherapy (Figure 1). HSCs are typically harvested from bone marrow or mobilised peripheral blood mononuclear cells (PBMCs) (Van Epps et al., 1994; Shpall et al., 1997). These are genetically modified using either an integrating viral vector or, more recently, by gene editing so that they express the gene of interest (Gomez-Ospina et al., 2019). This approach has recently been used in the clinic, with success, in the related condition metachromatic leukodystrophy (MLD) (Sessa et al., 2016).

HSCGT often makes use of a lentiviral vector, a member of the retrovirus family derived from the human immunodeficiency virus (HIV). Retroviruses are enveloped viruses that contain an RNA genome. The virus enters the cell *via* either direct fusion with the membrane or receptor-mediated endocytosis, it then “uncoats” and the viral RNA is reverse-transcribed to produce a proviral double-stranded DNA molecule. Viral proteins associate with the proviral DNA and facilitate nuclear entry and integration of the provirus into the host cell genome (Milone and O'Doherty, 2018). There are three genes that are essential for retroviral function, these are “gag” (encodes structural proteins), “pol” (encodes the reverse transcription machinery for the conversion of viral RNA to pro-viral DNA) and “env” (encodes the envelope glycoproteins) (Lewinski et al., 2006). Lentiviral vectors contain additional genes that amongst other things permit nuclear entry in non-dividing cells–thus making them ideal for stem cell transduction. 1st generation lentiviral vectors were produced by transfecting a suitable cell line (e.g., HEK293T or HeLa) with a plasmid containing the “gag” and “pol” sequences from the HIV virus and an envelope glycoprotein from the Vesicular stomatitis virus (VSV-G) (Daly and Chernajovsky, 2000). 2nd generation lentiviral vectors removed many of the accessory viral proteins that could potentially lead to viral replication in a treated patient (Naldini et al., 1996). Modern 3rd generation vectors are produced by splitting the “gag” and “pol” sequences from the “env” sequences, reducing the likelihood of producing replication-competent virus. Further improvements in safety came from the development of Self-Inactivating (SIN) lentiviral vectors, produced by introducing deletions into viral 3′LTR to completely ablate viral promoter activity from both the 5′ and 3′ LTR in integrated proviruses, thus significantly reducing the risk of off-target proto-oncogene activation (Zufferey et al., 1998; Bigger and Wynn, 2014), The production of lentiviral vectors for clinical application is reviewed in more detail by Milone and O’Doherty, 2018 (Milone and O'Doherty, 2018).

Lentiviruses are popular in gene therapy for their ability to stably integrate their genome into transduced cells. This makes them particularly popular in *ex vivo* stem cell gene therapy approaches as, once integrated, the lentiviral genome will propagate throughout subsequent generations (Milone and O'Doherty, 2018).

### Gene editing strategies

**FIGURE 2:**
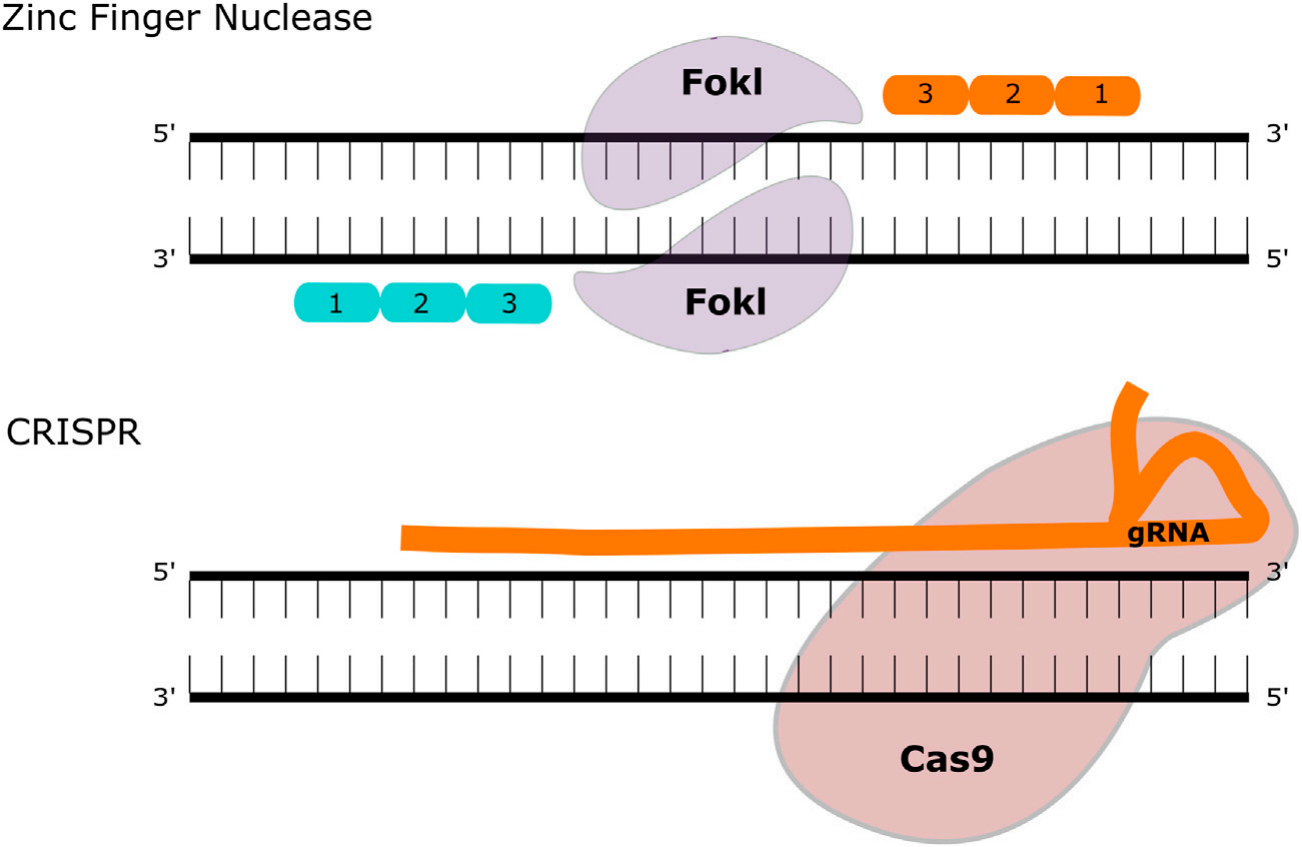
Gene Editing Methodologies for MPS. ZFNs contain two functional domains, a DNA binding and a DNA cleaving domain (comprised of the FokI nuclease). These create double-strand breaks that can be repaired with homologous recombination or non-homologous end joining. The CRISPR-Cas9 system utilises a guide (g)RNA and a Cas9 enzyme that introduces a double-strand break at the target site.

Gene editing is a technology that is rapidly gathering pace in the treatment of inherited disease, particularly since the development of the CRISPR/Cas9 system. The original gene-editing tools centred on meganucleases (naturally occurring endonucleases that can be engineered to target specific genetic loci), transcription activator-like effector nucleases (TALENs) and zinc-finger nucleases (ZFNs). All three systems rely on introducing a double stranded break to a targeted section of DNA, followed by directed or undirected repair Figure 2. The CRISPR/Cas9 system requires two main components: The RNA duplex containing a variable CRISPR RNA (crRNA) and a trans-activating crRNA (tracrRNA) that promotes DNA target recognition of the site of interest (collectively known as “guide” or gRNA) along with an RNA-guided DNA endonuclease (Cas9) that cleaves the DNA at that site. The potential of this system for gene-editing was demonstrated in prokaryotes (Gasiunas et al., 2012; Jinek et al., 2012) and then shortly afterwards in mammalian cells (Cong et al., 2013), reviewed in Broeders et al. (Broeders et al., 2020). Delivery of the gene editing machinery requires a vector to deliver the components, and this is usually AAV—either in two vectors or more recently in a single vector. To date, the majority of published gene editing studies in the MPS diseases have focused on MPSI. Both *ex vivo* and *in vivo* strategies have been described, using both viral and non-viral vector delivery.

### The challenge of the blood-brain-barrier in MPS disease

**FIGURE 3:**
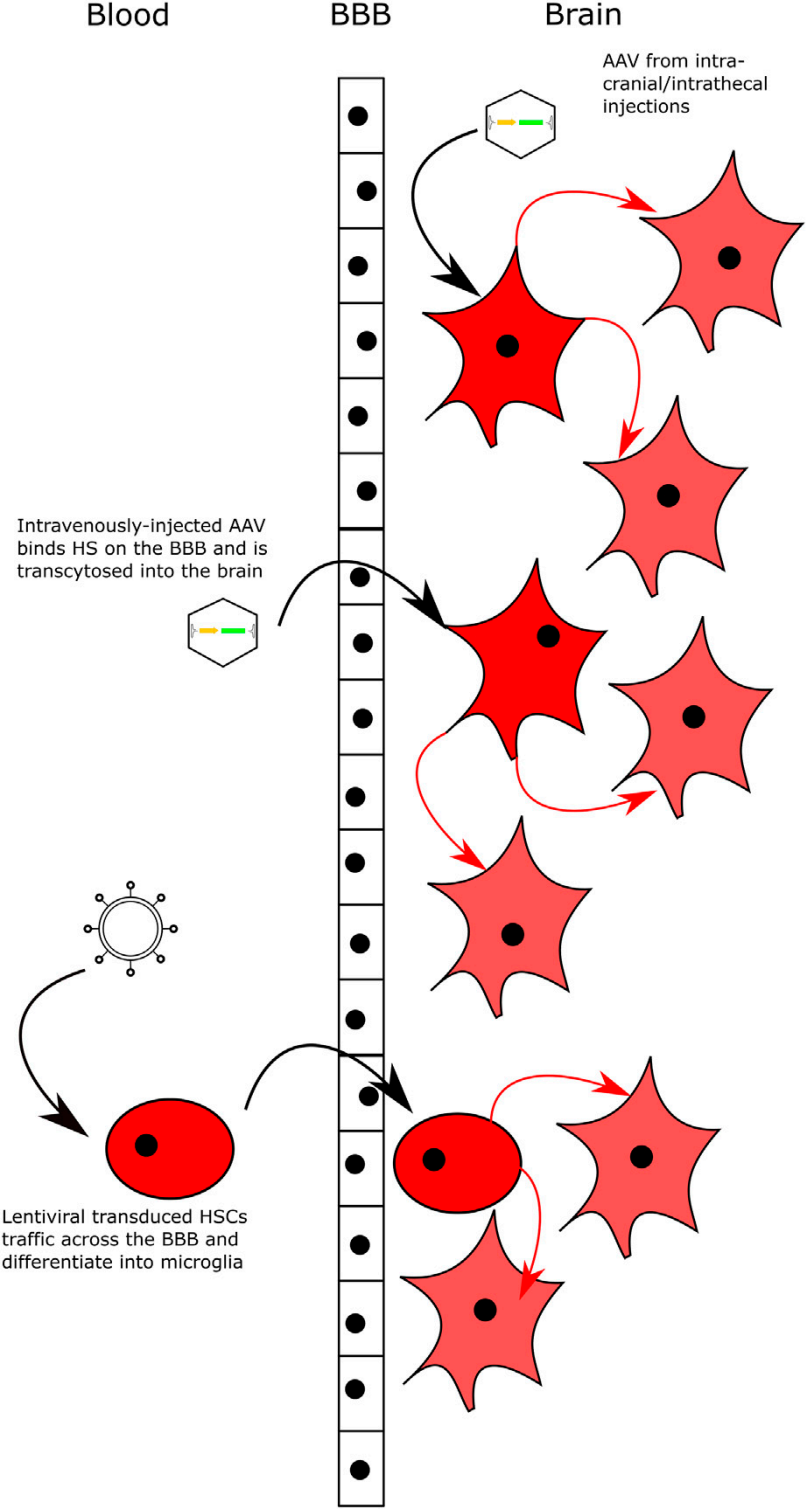
Delivering Gene Therapy to the CNS. Intracranial and intrathecal delivery of AAV vectors bypasses the BBB by direct injection into the CNS. Intravenous delivery of AAV serotypes that can bind HS on the surface of the BBB and are transcytosed across the BBB into brain. Lentiviral-transduced HSCs can cross the BBB and engraft in the brain following transplantation with myeloablative conditioning. All delivery methods lead to transduced neuronal cells that secrete high levels of the relevant enzyme and cross-correct neighbouring cells.

Arguably the greatest challenge in treating the neurological symptoms in MPS is delivering sufficient enzyme to the brain, a challenge greatly exacerbated by the BBB. Formed by endothelial cells lining the walls of vessels in the CNS, the epithelial cells of the choroid plexus and the arachnoid epithelium, the BBB is essential for regulating the CNS microenvironment (Abbott et al., 2010). Tight junctions formed between the various cells of the BBB, along with other molecular mechanisms (such as enzymatic degradation of molecules as they pass through the BBB) prevent the passage of many large molecules from the bloodstream into the brain (Abbott et al., 2006; Abbott et al., 2010). In MPS, the BBB poses a significant problem. ERT approaches, as mentioned above, can prove efficacious in treating pathology in peripheral organs (such as heart, lungs, etc) however the BBB prevents the entry of large enzymes, so the CNS remains poorly treated. Strategies to facilitate transport across the BBB have mostly exploited receptor-mediated transcytosis (RMT), with a particular focus on the transferrin receptor (TfR), insulin growth factor receptor (IGFR) and the low-density lipoprotein receptor (LDLR). ERT strategies include transferrin-receptor targeting antibodies, fused to IDS, for the treatment of MPSII (Boado et al., 2011; Sonoda et al., 2018), the receptor binding motif of Insulin-Dependent Growth Factor II (IGFII) fused to NAGLU for MPSIIIB (Kan et al., 2014) and the fusion of a plant lectin ricin peptide to IDUA for the treatment of MPSI (Ou et al., 2018). As mentioned previously, one of the main potential issues with this approach is the potential for antibody response to the enzyme. This could be exacerbated with the use of additional peptides as these could also been seen as antigens to a naive immune system. There have been attempts to modify gene therapy treatments with BBB-crossing peptides, in particular using peptides based on ApoE (LDLR-binding) in MPSI and MPSII (Wang et al., 2013; Gleitz et al., 2018). Both these studies utilised haematopoietic stem cell gene therapy strategies, where the immune system is “re-set” following conditioning and transplant. This could reduce the potential for anti-enzyme immune responses. Other studies have used the LDLR binding domain of the ApoB peptide to increase BBB-crossing following IV injection of AAV vectors in MPSIIIA (Sorrentino et al., 2013). Figure 3 illustrates current gene therapy strategies to deliver enzyme across the BBB.

## MPSI

MPSI is inherited in an autosomal recessive fashion and is caused by the deficiency of α-iduronidase (IDUA). Broadly, MPSI can be classified in three forms, an attenuated form known as MPSI-Scheie (MPSIS), an intermediate form known as MPSI-Hurler-Scheie (MPSIH/S) and a severe form known as MPSI-Hurler (MPSIH). Common to both forms are skeletal deformities, cardiac and pulmonary disease and upper airway obstruction, however neurological impairment is characteristic of Hurler and Hurler-Scheie (Pastores et al., 2007; Soliman et al., 2007; Muenzer et al., 2009a).

Proof-of-concept for AAV-mediated gene therapy in MPSI Hurler (MPSIH) has been demonstrated in several disease models. In the *Idua* ^−/−^ mouse model, direct intraparenchymal injection of AAV8, delivering IDUA, was shown to generate up to 40x wild-type mouse IDUA activity in the brain. This activity was sustained up to 10 months post-treatment (Wolf et al., 2011). This expression was able to normalise GAG and GM3 ganglioside deposits to wild-type levels compared to un-treated control mice. In Hurler dogs, intrathecal injections of AAV2/5 or AAV5/5, expressing IDUA, were able to reduce GAG deposits in the brain to near wild-type levels (Ciron et al., 2006; Ellinwood et al., 2011). There were also reductions in ganglioside storage (both GM2 and GM3) and detectable storage lesions. Other studies in the feline model of MPSI utilised intrathecal injections of AAV9 to deliver IDUA to the nervous system, producing elevated IDUA activity in brain and spinal cord at 6-months post-treatment compared to untreated control cats (Hinderer et al., 2014). Reductions in CNS lesions, GAG accumulation and ganglioside storage were also seen. Quantitative PCR (qPCR) demonstrated high numbers of vector copies throughout the brain, spinal cord and liver. An elevated antibody response to the therapeutic IDUA enzyme was seen, however this did not reduce the CNS correction. The same group demonstrated similar results in the dog model of Hurler. These dogs were tolerized to the human IDUA enzyme, at post-natal day 5, by hepatic gene transfer (using AAV8) of human IDUA, allowing analysis of the effects of gene therapy without the immune responses often seen when expressing human IDUA in dogs (Hinderer et al., 2015; Hinderer et al., 2016a). Subsequent intrathecal injections of AAV9-IDUA (at 1 month of age) produced consistent IDUA enzyme activity in the CSF over 6 months post-treatment (Hinderer et al., 2016a). This data led to the initiation of a phase I/II dose-escalation clinical trial (NCT03580083) to investigate the delivery of IDUA by AAV9 (RGX-111) in five patients with MPSI, interim analysis was presented at the WORLD conference in 2022 (Wang et al., 2022).

Early HSCGT approaches, in MPSI, utilised a γ-retroviral vector to transduce murine bone marrow cells, which were then transplanted into the *Idua* ^*−/−*^ mouse model (Zheng et al., 2003). Correction was seen in visceral organs such as the kidney, which was not corrected by transplanted wild-type bone marrow, however no correction was seen in the brain, possibly due to inadequate enzyme being delivered to the nervous system. This poor expression could be due to the use of whole body irradiation to condition the mice pre-transplant. The use of busulfan conditioning has been shown to greatly improve microglial engraftment post-transplant and is used clinically when performing HSCT in patients (Wilkinson et al., 2013). Low enzyme levels could also have been the result of poor expression from the γ-retroviral vector, with more recent approaches utilising 3rd generation lentiviral vectors (Wang et al., 2009). Lentiviral-mediated overexpression of IDUA in erythroid cells, *via* a lineage-specific promoter, was shown to normalise disease pathology in the liver, spleen and heart in the mouse model compared to wild-type littermates. Brain pathology was also improved, however not completely normalised. In order to improve delivery to the brain (and other organs), other groups have used ubiquitous promoters to drive IDUA expression post-transplant (Visigalli et al., 2010). Visigalli et al. report supra-normal levels of IDUA activity following transplantation of HSPCs that were transduced with IDUA LV. This expression facilitated correction of neurological pathology, such as reductions in the degradation of the Purkinje cell layer, and behavioural outcomes including improvements in memory (analysed in an open-field test). Skeletal pathology was also significantly improved by IDUA LV treatment, as was retinal thickness. This approach was shown to demonstrate little toxicity and tumorigenic potential following transduction of murine HSCs and a good biodistribution profile following transplantation of human CD34^+^ stem cells (transduced with IDUA LV) into NSG mice (Visigalli et al., 2016). The same group also investigated the effect of pre-existing immunity on the efficacy of HSCGT as many patients receiving ERT develop IDUA-specific CD8^+^ T Cells (Squeri et al., 2019). As MPSI patients are IDUA-deficient, the immune system views human IDUA as a foreign antigen. The researchers found that inducing an anti-hIDUA CD8^+^ T Cell response, using an intravenous injection of recombinant hIDUA in an incomplete Freund’s adjuvant (IFA), impaired engraftment of HSCs in *Idua* ^*−/−*^ mice. However data from HSCT studies in MPSI suggests that following transplant, immune tolerance is induced (median time ∼101 days post-transplant), an occurrence that could potentially happen following HSCGT (Saif et al., 2012). Following on from the data demonstrated in these studies, a phase I/II clinical trial is now underway (NCT03488394) and had enrolled eight patients by April 2019. Patients are receiving a conditioning regimen using busulfan, fludarabine and rituximab in an attempt to reduce ERT-induced anti-IDUA immunity (Poletti and Biffi, 2019). Interim results (∼2 years post-treatment) were reported in November 2021 and demonstrated a good safety profile, increases in IDUA expression in the blood along with decreases in urinary GAGs. CNS GAGs were also significantly reduced and patients demonstrated improvements in motor skills (6 out of eight patients within normal limits on Total, Gross and Fine Motor Quotient scores). Some improvements in longitudinal growth were also seen, suggesting that the therapy could have an impact on skeletal disease, another aspect of MPSI that is particularly difficult to treat. This indicates that cross-correction of non-haematopoietic cells is occurring. Seven patients were receiving ERT prior to transplant and the presence of anti-IDUA antibodies was detected in five of these patients at baseline. Encouragingly, these antibodies were undetectable 3 months post-transplant, indicating successful immune tolerance (Gentner et al., 2021).

The first gene-editing approaches in MPSI used ZFNs to introduce IDUA to the serum albumin locus in an attempt to increase expression. AAV2/8 was used to target the ZFN to the liver of wild-type C57BL/6 mice, producing a “protein-factory” (Sharma et al., 2015). Further studies were then performed in the MPSI mouse model where intravenous administration of AAV2/8-ZFN (at ∼1.5 × 1011 vector genome particles) and AAV2/8-hIDUA (at ∼2.0 × 1012 vector genome particles) was able to significantly increase IDUA activity in the blood, normalising GAG levels in somatic organs and improving behavioural deficits. GAG levels in the brain were improved but not normalised (Ou et al., 2019). The results of this study led to a phase I/II trial being initiated (ClinicalTrials.gov Identifier: NCT02702115). Interim results were presented at the WORLD Conference in 2019 and were able to demonstrate safety, however treatment did not appear to have any effect on increasing the levels of IDUA in the blood (Harmatz et al., 2019). Following on from this study, the researchers developed an improved approach based on CRISPR-Cas9, which demonstrated superior levels of IDUA activity in the plasma compared to the previous ZFN approach, as well as detectable IDUA activity in the brain in MPSI mice (Ou et al., 2020). However, IDUA enzymes are still low in the brain, with the highest levels being seen at very high doses of the two AAV vectors required to deliver the therapy (3 × 10^14^ vg and 5 × 10^13^ vg for AAV-IDUA and AAV-Cas9 respectively). These very high levels of AAV will be difficult to scale-up to human use with a high potential for immune responses against the viral capsid. CRISPR-Cas9 has also been used to edit the mutant IDUA sequence in human fibroblasts. Fibroblasts taken from patients with the most common MPSI mutation (W402X) were taken and a 20 bp gRNA targeted to the region within IDUA with the W402X mutation was used to correct the sequence. A reduction in lysosomal swelling was seen in the fibroblasts as was an increase in IDUA activity. However, only 4–7% of fibroblasts were successfully corrected and the increase in IDUA activity still only represented ∼6% of wild-type activity on average (de Carvalho et al., 2018). The same researchers then studied the effect of this treatment strategy *in vivo*. They used hydrodynamic injection to deliver CRISPR/Cas9 to MPSI mice. Significant increases in IDUA activity were seen in the blood, lung and heart, but not in the brain. The authors noted improved cardiac function, including heart dilation and hypertrophy in treated animals (Schuh et al., 2018). A recent study used CRISPR/Cas9 to introduce IDUA, under the control of a strong ubiquitous promoter, to the CCR5 safe-harbor locus, in an attempt to over-express IDUA in HSCs. Transplant of these cells into a NSG/IDUA mouse model demonstrated some efficacy such as reduced, but not normalised, neuroinflammation and clearance of GAG in the liver and spleen. GAG was not normalised in the brain however. Some behavioural deficits were improved, however some, such as working memory, were reduced in treated animals compared to un-treated NSG/IDUA (Gomez-Ospina et al., 2019).

Although gene editing approaches have potential for treatments in a range of diseases, current strategies are unlikely to be efficacious in MPS primarily due to the large amount of enzyme needed to treat lysosomal diseases. Under ideal circumstances direct gene editing (or replacement) would introduce one functional copy of the relevant gene into every cell of the body. Current gene editing protocols are not efficacious enough to achieve this given that we have an estimated 37.2 trillion cells in the human body (Bianconi et al., 2013). As a result, techniques that overexpress the gene of interest in HSCs are more likely to elicit a measureable effect on the condition, however gene editing of human HSCs is currently inefficient (Bak et al., 2018). The concept of using a safe harbour locus to overexpress IDUA is potentially a better approach to gene editing in MPSI, with some promising data in the MPSI mouse model (Gomez-Ospina et al., 2019; Ou et al., 2019), however poor outcomes in humans (Harmatz et al., 2019). A further issue with CRISPR gene editing, in particular, is the need for multiple vectors to deliver the template and other associated components. A recent study in MPSI mice looked at the use of a single AAV utilising the smaller size of different Cas9 homologues. Although increases in IDUA activity were seen in the liver, there was no effect on behaviour reported, liver GAGs failed to return to wild-type levels and there were no CNS outcomes reported (Ibraheim et al., 2021).

## MPSII

Unlike the other MPS conditions, which are inherited in an autosomal recessive manner, MPSII is an X-linked recessive disorder that primarily affects boys. Like MPSI, there are two broad classifications of MPSII, an attenuated form and a severe form (Hunter disease). The IDS deficiency results in a build-up of heparan sulfate and dermatan sulfate throughout the body resulting in a multi-systemic disease (Muenzer et al., 2009b). ERT for MPSII has shown efficacy in treating some pathology however the CNS, heart and respiratory systems remain poorly corrected. HSCT is occasionally given to very young patients (<2) but has limited effect on the CNS in MPSII, possibly due to the enzyme levels delivered by engrafting microglia being too low to facilitate correction (Bigger and Wynn, 2014). HSCT may have more efficacy when used to treat patients prior to the onset of developmental delays and would be worthwhile pursuing, however overall correction still remains poor (Tanaka et al., 2012).

The majority of gene therapy approaches in MPSII focus on AAV as a delivery vector. Intravenous injections of MPSII mice with an AAV2/8 vector expressing human IDS under the control of a liver-specific promoter was able to generate high levels of IDS in the blood up to 7 months post-treatment. The increase in IDS activity was able to normalise GAG levels in the urine and somatic tissues but the brain remained poorly corrected (Cardone et al., 2006). This is likely due to the BBB preventing the crossing of the enzyme into the brain. Further studies have evaluated the use of AAV2/9 to deliver IDS to MPSII mice. One study used intracisternal injection of AAV2/9-IDS and demonstrated correction of neuroinflammation (demonstrated by a reduction of astrocytosis and microgliosis, as analysed by GFAP and ILB4 staining respectively) and GAG content in the brain (analysed by a Blyscan Glycosaminoglycan Assay). Multiple somatic organs had normalised GAG content following treatment, despite having no detectable vector genomes, suggesting cross-correction from the circulation (Motas et al., 2016). Other groups have used intracerebroventricular administration of AAV2/9-IDS to MPSII mice, demonstrating similar results (Hinderer et al., 2016b; Laoharawee et al., 2017). These have led to the technology being licenced by Regenxbio and the commencement of a phase I/II clinical trial (ClinicalTrials.gov identifier: NCT03566043). Interim results were presented at WORLD in 2022, with decreases in urinary and CSF GAG accumulation and improvements in skill acquisition shown (Harmatz et al., 2022).

Proof-of-concept for haematopoietic stem cell gene therapy for MPSII has been demonstrated by two groups (Wakabayashi et al., 2015; Gleitz et al., 2018). Wakabayashi and colleagues, at The Jikei University School of Medicine in Tokyo, Japan, used a second generation lentiviral vector to overexpress IDS in murine HSCs. Following transplant, *Ids* ^*−/−*^ (MPSII) mice demonstrated partial reductions in GAG storage in the brain, liver and heart. Treated mice were also found to have improvements in short-term memory performance and a reduction in LC3-II, a marker of autophagy, compared to untreated controls. This data indicates that HSCGT has potential as a therapy for MPSII. Further work from this group in 2020 also demonstrated reductions in bone mineral density and, bone thickness and bone surface area in treated MPSII mice compared to controls (Wada et al., 2020). As skeletal dysplasia is a key symptom in MPSII, the ability to effectively treat bone tissue is important. A modified HSCGT strategy was published by Gleitz and colleagues in 2018 (Gleitz et al., 2018). In this study MPSII mice were transplanted at 2 months with HSCs transduced with a lentivirus expressing a modified human IDS enzyme. The enzyme was tagged with an ApoEII peptide sequence (LV.IDS.ApoEII), designed to improve uptake of peripheral enzyme across the BBB. MPSII mice showed improvements in behavioural outcomes (working memory and balance) when treated with LV.IDS or LV.IDS.ApoEII, with the latter the only treatment to fully normalise both parameters back to wild-type performance. The width of the zygomatic arches, humerus and femur were all normalised with both LV.IDS and LV.IDS.ApoEII. Mice were culled at 6 months post-treatment to analyse biochemical parameters. Increases in IDS activity were seen throughout the body. Interestingly, although there was no increase in brain IDS activity in the LV.IDS.ApoEII treated group compared to the LV.IDS treated group, LV.IDS.ApoEII was the only treatment to fully correct microgliosis, astrocytosis and lysosomal swelling in the brain as well as cognitive working memory on the Y-maze. HS storage and sulfation pattern in the brain was also normalised to wild-type by LV.IDS.ApoEII but not by LV.IDS. This suggests that improvements in trafficking enzyme across the BBB may not be the only mechanism by which ApoEII is acting.

Gene editing approaches have been trialled in MPSII patients. The same ZFN approach described above for MPSI was utilised, with similar results in MPSII mice with GAG reductions seen throughout the body, with little correction of the brain (Laoharawee et al., 2018; Muenzer et al., 2019). A clinical trial was initiated from this work (ClinicalTrials.gov Identifier: NCT03041324) and the interim results were presented at the WORLD 2019. Like the related MPSI clinical trial, the results demonstrated that the approach was safe and well-tolerated, however, to date, little efficacy was shown and efficacy data has not been published (Muenzer et al., 2019).

## MPSIII

There are several subtypes of MPSIII (also known as Sanfilippo disease), each caused by the deficiency of a different enzyme. Sulfamidase (SGSH) in MPSIIIA, N-acetylglucosaminidase (NAGLU) in MPSIIIB, Acetyl-Coα-glucosaminide acetyltransferase (HGSNAT) in MPSIIIC and N-acetylglucosamine 6-sulfatase (GNS) in MPSIIID. Common to all subtypes is the progressive mental decline, hyperactivity and dysmorphic facial features (Wraith, 2002; Jansen et al., 2007; Ruijter et al., 2008; Valstar et al., 2008; Heron et al., 2011; Valstar et al., 2011; Wijburg et al., 2013; Shapiro et al., 2016) with relatively mild somatic involvement.

AAV-mediated gene therapy has shown promising results in the mouse models for MPSIIIA, MPSIIIB, MPSIIIC and MPSIIID. 5-week old, pre-symptomatic MPSIIIA mice undergoing intracerebroventricular delivery of AAV2/5 expressing SGSH and SUMFI (an enzyme involved in the post-translational modification of SGSH-AAV2/5.CMV.SGSH.IRES.SUMFI) was able to reduce lysosomal storage, inflammatory markers and microgliosis in the MPSIIIA mouse. Improvements were also seen in motor, cognitive and memory deficits as determined by gait measurement, open-field tests and the water maze (Fraldi et al., 2007). Based on the results of this study, the treatment was licensed to Lysogene, Inc. and a phase I/II clinical trial (NCT01474343) was started, with four patients (ranging from 6 months old to 3 years 1 month old at inclusion) undergoing intracranial injection with AAVrh10-SGSH-IRES-SUMFI. Three patients were considered to be clinically stable at the end of the year follow-up and one patient was considered to have shown improvement, however some increases in brain atrophy were observed in two patients. Also a decrease in fine motor movements was seen in patients 2 and 3. To the author’s knowledge there has been no update published to date on these patients (Tardieu et al., 2014). A further pre-clinical study using a similar vector modified with a different promoter (replacing the phosphoglycerate kinase or ‘PGK’ promoter with the chicken beta actin/CMV composite (CAG) promotor) and the removal of SUMFI. This vector showed further improvements in enzyme expression in the brains of MPSIIIA mice as well as, more recently, healthy non-human primates and dogs (Gray et al., 2019; Hocquemiller et al., 2020). The therapy, using the CAG-SGSH vector, delivered *via* intra-cerebral injection, recently entered phase II/III clinical trial (Clinical trial identifier: NCT03612869) recruiting patients above the age of 6 months old with a confirmed diagnosis of MPSIIIA *via* genotyping. Proof-of-concept data for this trial was published in 2020, where the CAG-SGSH vector was administered to mice (to examine long-term therapeutic efficacy), dogs and cynomolgus monkeys (both to study distribution). Other studies using an AAV9 vector demonstrated correction of lysosomal swelling and GAG storage throughout the body (brain, heart, liver, lung, spleen and kidney) of MPSIIIA mice. Complete correction of microgliosis and astrocytosis were also seen in the brain, along with improvements in locomotor function. These results were also repeated in a dog model (Haurigot et al., 2013). Following on from these results, a clinical trial was started in Europe (2015–000359–26—European Clinical Trial Register).

Another potential route of administration is intravenous injection, as certain AAV serotypes have the ability to cross the BBB (Zhang et al., 2011). IV administration of AAV1 and AAV8 expressing *SGSH* under a liver-specific promoter, was able to drive SGSH expression in the livers of MPSIIIA mice, generating high circulating SGSH in the bloodstream (as detected in serum) (Ruzo et al., 2012). The IV administration of a self-complementary AAV vector (scAAV–described in more detail in “Limitations of Current Approaches”) expressing SGSH (scAAV9-U1a-hSGSH) was able to normalise behavioral parameters and lifespan in mice treated at 3 months of age, and partially normalise the same parameters in older mice (treated at 6 months) (Fu et al., 2016). Recently this treatment was also shown to reduce HS levels in the urine, CSF and brain in MPSIIIA mice in both 3 months-treated and 6 months-treated cohorts (Saville et al., 2020). These results led to the initiation of two clinical trials, the first trialing IV administration of scAAV9-U1a-hSGSH in MPSIIIA patients under 2 years of age (or over 2 years but with a Developmental Quotient of >60) and a second clinical trial of the same treatment in patients with more advanced disease (Developmental Quotient <60). These are commercial trials, led by Abeona Therapeutics Inc., clinical trial identifiers NCT02716246 and NCT04088734 respectively. Updates were presented at the WORLD conference in 2022, with younger patients (<30 months of age at time of treatment) showing improvements in neurocognition, systemic biomarkers and other disease parameters (Flanigan et al., 2022). This programme has now been licenced to Ultragenyx pharmaceuticals.

AAV-mediated gene therapy delivering NAGLU to MPSIIIB mice has shown efficacy in pre-clinical studies. Intracranial delivery of AAV2/2 and AAV2/5 vectors expressing NAGLU was able to generate high levels of NAGLU activity in the brain of MPSIIIB mice (with AAV5 demonstrating higher activity and better distribution throughout the brain than AAV2). Some correction of disease parameters was demonstrated but the restriction of enzyme to the brain meant that no somatic correction was seen (Cressant et al., 2004). A study of AAV2/5 delivery of NAGLU, *via* intracerebral injection, in the MPSIIIB dog model demonstrated some efficacy in increasing NAGLU activity in the brain and reducing the storage of GAGs and GM2 gangliosides (Ellinwood et al., 2011). The authors also demonstrate that the absence of immunosuppression along with AAV delivery eliminates NAGLU expression in treated animals. However, given the low “n” number in several groups (*n* = 2 in the wild-type control group and *n* = 2 in the AAV-NAGLU treated without immunosuppression group) these results should be interpreted with caution. A phase I/II clinical trial was started based on these results and reported improvements in neurocognitive measures (assessed by Brunet-Lezine revised test and the Vineland Adaptive Behaviour Scale) with greater improvement in younger patients However, improvements were small and even the youngest patient failed to achieve scores that followed an “un-affected” child’s progression (Tardieu et al., 2017). A 5.5 years follow up reported lumbar CSF NAGLU at 18% of normal, but also a T cell response against NAGLU enzyme and a neuropsychological profile that is consistent with natural history in three of four patients. One patient showed a significantly better cognitive outcome than natural history but not within the normal range (Deiva et al., 2021). Intracisternal delivery of NAGLU *via* an AAV2/2 vector was able to facilitate CNS correction in MPSIIIB mice, including improved cognitive function (measured in the water maze) and reduced GAG storage and lysosomal swelling (Fu et al., 2010). Interestingly, the researchers show limited NAGLU expression in somatic tissues with an intracisternal injection of AAV2/2-NAGLU, compared to the increased SGSH expression seen in somatic tissues following similar intra-CSF approaches for MPSIIIA (Haurigot et al., 2013). A further study looked at the intravenous administration of an AAV2/9 vector to MPSIIIB mice. IV administration of AAV2/9-CMV-hNAGLU was able to correct lysosomal storage in the brain, increase lifespan and correct cognitive and motor function in MPSIIIB mice compared to untreated controls (Fu et al., 2011). A phase I/II clinical trial was initiated, sponsored by Abeona Therapeutics (NCT03315182) but has since been terminated.

Proof-of-concept for *ex-vivo* HSCGT has been shown in the mouse models of MPSIIIA and MPSIIIB (Langford-Smith et al., 2012; Sergijenko et al., 2013; Holley et al., 2018). Overexpression of SGSH in HSCs was able to correct hyperactivity and other behavioural pathology in the MPSIIIA mouse model. The lentiviral vector utilises a myeloid promoter (CD11b) to increase expression in microglia. Treatment of 2 months old, busulfan-conditioned MPSIIIA mice led to brain SGSH activity at ∼11% of wild-type levels at 6 months post-treatment. This level of expression led to complete correction of neuropathology and hyperactivity. Recent pre-clinical proof-of-concept in human CD34^+^ stem cells has also been completed (Ellison et al., 2019). This HSCGT strategy for MPSIIIA has been licenced to Orchard Therapeutics and entered clinical trial in late 2019 (NCT04201405). Interim results demonstrating excellent biochemical correction have been presented at the WORLD conference in 2021, but it is still too early to comment on neuropsychological outcomes (Kinsella et al., 2021). In MPSIIIB, HSCGT with a lentivirus overexpressing NAGLU under the control of the CD11b promoter (LV.NAGLU) was able to generate supranormal levels of NAGLU activity throughout the body of MPSIIIB mice, including ∼13% of wild-type expression in the brain. This increased activity facilitated the correction of lysosomal swelling, microgliosis, astrocytosis and GAG storage. The hyperactivity seen in MPSIIIB mice was also corrected as demonstrated by open-field tests (Holley et al., 2018).

The two remaining MPSIII subtypes are less studied in terms of gene therapy. In MPSIIIC treatment is more complicated than other MPS subtypes as the HGSNAT enzyme is localised to the lysosomal membrane and is not secreted (Durand et al., 2010). This means that the principal of cross-correction does not apply in MPSIIIC and, therefore, HSCGT strategies are unlikely to have a significant effect. The most promising gene therapy intervention is likely to be a direct injection of AAV into the brain. Recent preclinical evaluation of AAV9 and AAV-TT (“True-type”—a novel capsid based on AAV2) in the MPSIIIC mouse model demonstrated correction of neuropathology and correction of working memory deficits with the novel AAV-TT vector demonstrating improved widespread expression throughout the brain of treated mice (Tordo et al., 2018). MPSIIID is caused by the deficiency of N-acetylglucosamine 6-sulfatase (GNS) and a mouse model was recently described by Roca and colleagues (Roca et al., 2017). To the author’s knowledge there has only been one study in this mouse model utilising a gene therapy approach to rescue the disease phenotype. Roca and colleagues used intra-CSF injection to deliver an AAV2/9 vector expressing GNS to MPSIIID mice. Complete normalisation of brain GAG was shown as where significant reductions in brain lysosomal swelling and neuroinflammation. Given that GNS is secreted in a similar way to SGSH and NAGLU, a HSCGT approach may also be feasible but is yet to be tested.

## MPSIV

MPSIVA is caused by a lack of N-acetylgalactosamine-6-sulfate sulfatase (GALNS) due to mutations in the GALNS gene (Khan et al., 2017; Peracha et al., 2018). Although attempts at producing a viable gene therapy for MPSIVA have been undertaken, development of therapies are complicated by the apparent lack of appropriate animal models. *Galns* ^*−/−*^ (MPSIVA) mice demonstrate KS accumulation throughout the body, however the skeletal dysplasia seen in MPSIVA patients is absent (Tomatsu et al., 2003). Tomatsu and colleagues recently published a liver-directed AAV gene therapy that generated up to 19-fold greater than wild-type expression of GALNS enzyme in the plasma of MPSIVA mice and resulted in reductions in KS storage throughout the body over untreated KO mice (Sawamoto et al., 2020). More recently, a rat model was described by Bertolin and colleagues in which disease progression far more closely resembles the human skeletal disease (Bertolin et al., 2021). Treatment of this model with an IV injection of AAV9-*Galns* (using a strong, ubiquitous “CAG” promoter) was able to rescue the phenotype in rats. To the author’s knowledge no clinical trials have been initiated using gene therapy in MPS IV.

## MPSVI

Further use of gene therapy in the MPS diseases has been demonstrated in MPSVI. MPSVI, also known as Maroteaux-Lamy Syndrome, is caused by mutations in ARSB, leading to a deficiency in Arylsulfatase B (Tomanin et al., 2018). Unlike MPSI, II and III, MPS VI patients do not show neurological involvement. Clinical features of MPSVI can include growth retardation, dysostosis multiplex, cardio-respiratory complications and ocular manifestations such as corneal clouding (Valayannopoulos et al., 2010). There are many animal models of MPS VI, including mice, rat and cat models (Yoshida et al., 1993a; Yoshida et al., 1993b; Haskins, 2007) (refs), some demonstrating more severe phenotypes than others (Crawley et al., 1998). Intravenous injections of AAV2/8-ARSB in cat and rat models of MPSVI generated therapeutic levels of Arylsulfatase B which were able to clear GAG storage, reduced inflammation and corrected skeletal pathology. Some rats developed high levels of neutralising antibodies to the AAV vector which had no effect on disease correction. This immune response was absent in cat models treated with the same vector (Tessitore et al., 2008). Pre-clinical toxicology studies using a clinical-grade GMP vector in a MPSVI mouse model demonstrated no detectable toxicity (Ferla et al., 2017). A phase I/II clinical trial (MEUSIX) was initiated in 2017 and is ongoing (NCT03173521). Interim results presented in 2021 showed a good safety profile with sustained transgene expression 12 months post-surgery, however raising GAG levels were also seen at all doses (Brunetti-Pierri et al., 2021). To the author’s knowledge HSCGT strategies in MPSVI have not been published.

## MPSVII

MPSVII (Sly Syndrome) is caused by the deficiency of β-glucuronidase (GUSB) and has both neurological and multisystem involvement. To the author’s knowledge, there have been no clinical trials involving gene therapy for MPS VII. Several pre-clinical studies have been published using AAV gene transfer to treat animal models of MPSVII. Early studies used retroviral-mediated HSCGT to treat the liver and spleen of MPS VII mice with an intravitreal injection of adenovirus to treat the eye (Sands et al., 1997). Further studies using IV injections of AAV-GUSB, into neonatal MPS VII mice, demonstrated detectable increases in GUSB enzyme activity for 1 year post-treatment as well as alleviating disease throughout the body (Daly et al., 1999; Daly et al., 2001). More recent studies have used AAVrh10 to deliver GUSB to the CNS of MPS VII mice, resulting in widespread transduction throughout the CNS and correction of behavioural phenotypes (Pages et al., 2019). Despite a large number of pre-clinical studies being published, at the time of writing, no clinical trials have been initiated.

## Limitations of current approaches

AAV gene therapies for MPS are arguably the most well researched with numerous pre-clinical and clinical studies published. Limitations include the high cost of manufacturing related to the inefficiency of three-vector packaging systems and the amounts needed to treat organs such as the brain. These have been reviewed more extensively elsewhere (Wang et al., 2019). The cost of manufacturing plus the extensive amount of money needed to put AAV therapies through clinical trial leads to a high cost per-patient of approved products. For example, the approved AAV therapies Glybera (lipoprotein lipase deficiency–LPLD), Luxturna (Leber Congenital Amaurosis–LCA) and Zolgensma (Spinal Muscular Atrophy–SMA) had prices ranging from ∼$425,000 to ∼$2.48 million per patient at launch between 2012 and 2021.

Intraventricular, intracisternal magna and intrathecal injections of AAV to target the brain typically require large amounts of virus to elicit an effect. Intravenous injections of AAV typically require very high amounts of virus to elicit a therapeutic effect. In clinical trials for MPSIIIA and MPSIIIB, doses ranged up to 5 × 10^13^ vg/kg and 1 × 10^14^ vg/kg respectively (NCT02716246 and NCT03315182) for example. Even in very young patients this can mean 10–20 kg weights with total doses in the 5 × 10^14^ to 2 × 10^15^ vg range per patient. Intraparenchymal delivery uses less vector than IV, however they are still typically high, with clinical trials for MPSIIIA and MPSIIIB using between 7.2 × 10^11^ vg and 4.0 × 10^12^ vg per patient (NCT01474343 and NCT03300453). However, given the poor behavourial outcomes from these trials, it is likely that more vector will be needed to see a therapeutic effect (Tardieu et al., 2014; Tardieu et al., 2017). Amounts of AAV used in ICV and IT injections are much greater, normally between 1.0 × 10^10^/g brain mass and 2.0 × 10^10^/g brain mass as seen in trials for MPSI and MPSII (NCT03580083 and NCT03566043). If we consider that the human brain is approximately 1200 g, even in 1 year olds, these doses are approximately 1.2 × 10^13^–2.4 × 10^13^ vg/patient.

A further limitation of AAV gene therapy is the potential for immune responses against the AAV capsid. Human exposure to AAV in the environment has led to high seroprevalence of neutralising antibodies in people across the world, with one study from Calcedo and colleagues showing that the prevalence of neutralising antibodies to AAV2 was consistently higher than other serotypes and, in Africa, reaching over 50% prevalence (Calcedo et al., 2009). High neutralising antibody expression could lead to a reduction in therapeutic gene expression and potentially large immune responses (Fu et al., 2017). Overall, pre-existing neutralising antibody levels differ between serotypes but could be present in as low as 3% (AAV5) to as high as 100% (AAV2) by some estimates (Louis Jeune et al., 2013). In terms of MPS therapy, AAV9 is most commonly used. Boutin and colleagues estimated that neutralising antibodies for AAV9 were present in roughly 47% of individuals tested. Other vectors commonly used in MPS therapy are AAV5 (estimated at a range of 3.2%–50% of individuals) (Erles et al., 1999; Boutin et al., 2010) and AAVrh10 (estimated at ∼21%) (Thwaite et al., 2015). Mingozzi et al. also demonstrated a high proportion of cellular immunity to AAV2 in 17 healthy volunteers with eight showing AAV capsid-specific CD8^+^ T cells and nine having AAV capsid-specific CD4^+^ T cells. Strategies to circumnavigate neutralising antibodies may prove crucial to the future success of AAV gene therapies, for example the use of IgG-degrading enzymes have shown some promise in digesting neutralising antibodies *in vivo* (Elmore et al., 2020). Other barriers to AAV use include the relatively small amount of DNA that can be packaged into an AAV capsid (∼4.7 kb) and the delay in gene expression due to second strand synthesis. The former of these issues can be overcome with the use of “dual” vector systems if the gene of interest is too large to be packaged alongside a promoter and other regulatory elements (McClements and MacLaren, 2017). The rate-limiting step of second-strand synthesis can be overcome using a “self-complementary” vector, whereby an inverted repeat genome is packaged as double-stranded (ds) DNA, removing the need for second-strand synthesis (McCarty, 2008). However, it is unclear how removing the second strand synthesis step would benefit MPS treatment and some studies suggest issues with scAAV such as an increase in inflammatory markers (Martino et al., 2011).

The best route of administration of AAV is still not clear, and will likely differ depending on disease subtype. Direct delivery to the target organ (such as *via* ICV) produces robust expression but involves an invasive surgery and often leads to localised expression of the therapeutic enzyme. Other delivery methods, such as IV, have the advantages that they target multiple organ systems however enzyme expression in each organ is lower than with a direct delivery and there is a significant possibility for immune responses against the vector and the transgene. Also, our studies (and others) in MPS mice have suggested that GAGs in the brain need to be reduced by 50–70% to see positive behavioral outcomes, a bar that many clinical AAV studies have not hit.

To address immunological concerns from vector delivery or pre-existing capsid seropositivity a range of immunosuppressant regimens have been used. For example mycophenolate mofetil and tacrolimus (Tardieu et al., 2014) or a tapering course of prednisolone (NCT02716246); however it is not clear that there is a scientific basis behind the choice of immunosuppressive regimens. This needs to be addressed.

*Ex-vivo* HSCGT also has great potential in the treatment of MPS disease, having already proved revolutionary in similar lysosomal diseases and with several clinical trials now underway. The use of autologous cells reduces the likelihood of graft vs. host disease and completely resets the patient’s immune system, making adverse T Cell responses less likely, as demonstrated in the recent MPSI trial (Gentner et al., 2021). Also, as the virus is used to transduce cells *ex vivo*, the viral vector is not introduced to the patient, further reducing the likelihood of immune responses. In HSCGT, selecting the correct conditioning regimen is crucial to optimise engraftment of cells in the brain and other hard to treat organs. Current conditioning regimes use myeloablative chemotherapy, in particular Busulfan, which can cause severe side-effects such as alopecia, anemia, intestinal damage and, in some cases, malignancy (Patel and Tadi, 2021). Other conditioning options include treosulfan, however this does not cross the BBB and shows reduced brain engraftment compared to busulfan (Capotondo et al., 2012). The development of other conditioning regimens that achieve high levels of engraftment of transplanted cells whilst limiting side-effects will be crucial to moving HSCGT forward. The use of lentiviral vectors has several advantages when compared to AAV, including more long-term gene expression and fewer scale-up issues, however they come with the potential issue of insertional mutagenesis, potentially leading to oncogenesis. This was seen in two separate clinical trials (one in London, another in Paris) in patients with X-SCID, where HSCGT was able to restore immune function in patients however, several years later a subset of patients would go on to develop leukaemia (Cavazzana-Calvo et al., 2000; Hacein-Bey-Abina et al., 2002). This was later discovered to be due to the insertion of the lentiviral cassette into a proto-oncogene called LMO2 (Hacein-Bey-Abina et al., 2002). The mechanisms surrounding this insertional mutagenesis event are reviewed in more detail by Kohn et al., 2003 (Kohn et al., 2003). The use of SIN vectors removes this viral promoter activity, reducing the activation of proto-oncogenes downstream from the insertion site. The switch to SIN lentiviral vectors also improves safety by reducing the growth selection of transduced HSCs to ∼2 days in culture rather than ∼5 (Bigger and Wynn, 2014) and has significantly reduced the risk of oncogenesis from viral integration to extremely low. *Ex vivo* gene therapy is also expensive with prices for Strimvelis (ADA-SCID) and Libmeldy (Metachromatic leukodystrophy) at £594,000 and £2.8 million respectively.

Gene editing is a relatively new concept compared to the other gene therapy modalities mentioned in this review, however in MPS diseases these are unlikely to be sufficiently efficacious in enough cells to significantly impact disease. In most MPS subtypes (except MPSIIIC), secretion and cross-correction of the disease is critical, therefore either a great enough number of cells must be successfully modified or the subset of cells successfully modified will have to over-express the enzyme. The use of the safe-harbor locus to insert a cassette designed for this purpose may prove to be the most efficacious (Gomez-Ospina et al., 2019). The use of two or even three vector systems to deliver CRISPR Cas9 to cells is not going to be as efficient as using a single vector and may have been the reason behind poor liver expression in patients. The CRISPR Cas9 system can be packaged into one AAV, using different homologues of Cas9. However, potential problems with constitutive expression of Cas9 *in vivo* have been demonstrated in recent studies and will be need to be overcome before this becomes a more realistic option (Ibraheim et al., 2021). *Ex vivo* gene editing into stem cells at a safe harbour locus will always be the more appealing approach, but to date clinical experience with ZFNs has been disappointing and no clinical studies using CRISPR-Cas9 have been reported. Other issues with AAV6 delivered gene editing in HSCs, potentially leading to poor repopulation and engraftment following transplant, also need addressing before this becomes a viable option (Romero et al., 2019).

## Conclusion

Gene therapy is a promising treatment avenue for patients suffering with MPS. It seems that a few fundamental principles are applicable to gene therapy in MPS, amongst which are a need for over-expression of the enzyme, to allow for the relatively low number of cells that are typically transduced by gene therapy vectors, as well early treatment of patients to reduce the damage done in the brain and other organ systems prior to treatment. Future improvements that will be critical for success include better regulated enzyme expression from vectors and more targeted approaches for getting enzyme to specific organs. As therapies are developed and gain market approval, it will become increasingly important to ensure that adequate newborn screening platforms are in place to allow identification of patients and allocation to therapies as early in life as possible. There is no doubt that earlier treatment improves outcomes especially where the brain is involved. Gene and cell therapies are currently very expensive treatments for patients. Reducing development and production costs are critical next steps for the sector.
